# The Design of the Quadrangle

**Published:** 1918-09-28

**Authors:** 


### THE NURSERY SCHOOL.

### The Design of the Quadrangle.

In 1909 Miss Margaret McMillan made an experiment by starting at Deptford a school clinic with ambitious aims, which have been quietly pursued ever since. The clinic is in fact a nursery school, and we are not sure that a family school would not be a more accurate name for it. Near the school is a baby camp, and to the camp and the school come not only babies and elder children and, on occasion at least, their mothers, but probationers and teachers too. The camp is a house with one of those odd pieces of "waste" ground that are often to be found in London's densest centres of population, and here the children eat and sleep and bathe to the average number of fifty, of whom twelve are under a year old. The mother who is suckling her infant is encouraged to come, and as her child grows up advantage is taken of its curiosity to bring it into the open-air shed, where first lessons are given and over which a head-mistress presides.

The probationers learn the elements of nursing as well as those of teaching, and receive a two years' training before becoming eligible for the certificate granted to Rachel McMillan nurse-teachers. Probationers, who attend for one month in the first instance, may be anything from sixteen to thirty years old. The fees which they must pay amount to £40 a year. Needlework lessons form part of their training, so that they may learn to make strong cotton clothes of good colour and design, with no taint of ugliness. At the end of their training the probationers are deemed to be capable of looking after all children up to the age of seven, and, we hope, of assisting to conduct new clinics of the same class in new centres. At all events, they acquire the rudiments of health and school visiting and of child welfare work. The principal object in view has been to provide an open-air life for the city child without the attendant dangers of the street; in short, to allow it to be out of doors and at home at the same time. Of course it is a thoroughly bad system which leads us to provide for the poor that which they should be able to provide for themselves, for it means their regimentation, the destruction of family life, and a wretched amount of more or less officious interference. Fundamentally, child welfare work is not a remedy for, but a part of, the economic disease. As things are, we move inevitably in a vicious circle.

The open-air home described above presents the economic difficulty in the form of an architectural problem. If all the families of Deptford were self-supporting and prosperous, their children would still have to choose between a wretched dwelling and the street. In describing Miss McMillan's Nursery School, a writer in a recent number of the *Times Educational Supplement* said that what is wanted is the (impossible) experiment of "rebuilding large districts of our towns in the form of hollow squares." This at once calls up a picture of the Cambridge Court and the Oxford Quadrangle. It does more, it reminds us that the most beautiful form which a house can take is that of low buildings round a central court or piazza, and because this form is the most beautiful it is also the most healthy and convenient. Private houses which cannot run to a whole quadrangle can adopt the L shape, which suggests it, and are most beautiful when they do so. Such buildings, which deliberately occupy a considerable space of ground, imply the ease which the possession of space can give. They do away with high buildings, which are ugly, and for this reason unhealthy and poverty-stricken. We instinctively say that people who live in high houses are in straitened circumstances. So they are. This excursion is useful in reminding us that rickets, dental caries, and tuberculosis are largely diseases of poverty, and until that vast problem is tackled we shall continue to propagate them faster than to furnish their remedy.

The Education Act, which advocates the establishment of nursery schools, has at all events some existing models to work from. In the belief that further grants for them will be forthcoming, the committee of the Birmingham Nursery Schools' Association opened last January a third nursery school in the Memorial Hall, Farm Road, Sparkbrook. It is to be hoped that, after the war, the new schools which we may then expect will be linked up with the town-planning movement, and that we shall see in the buildings which will then arise a recognition of the quadrangle as the proper architectural design for them.

